# Directly or Indirectly? The Role of Social Support in the Psychological Pathways Underlying Suicidal Ideation in People with Bipolar Disorder

**DOI:** 10.3390/ijerph19095286

**Published:** 2022-04-26

**Authors:** Rebecca Owen, Steven H. Jones, Robert C. Dempsey, Patricia A. Gooding

**Affiliations:** 1 Division of Psychology & Mental Health, School of Health Sciences, Faculty of Biology, Medicine and Health, University of Manchester, Manchester M13 9PL, UK; rebecca.owen@merseycare.nhs.uk (R.O.); patricia.a.gooding@manchester.ac.uk (P.A.G.); 2 Mersey Care NHS Foundation Trust, Prescot L34 1PJ, UK; 3 Spectrum Centre for Mental Health Research, Faculty of Health and Medicine, Lancaster University, Lancaster LA1 4YT, UK; s.jones7@lancaster.ac.uk; 4 Department of Psychology, Faculty of Health and Education, Manchester Metropolitan University, Manchester M15 6BH, UK

**Keywords:** suicide, suicidal experiences, psychosocial, bipolar, mediation

## Abstract

Contemporary theories of suicide, such as the Schematic Appraisals Model (SAMS), hypothesize that negative perceptions of social support are implicated in the pathways to suicidal experiences. The SAMS predicts that perceived social support influences suicidal ideation through appraisals of defeat and entrapment. However, such pathways have not been investigated in people who have bipolar disorder. This prospective four-month study tested the influence of perceived social support on later suicidal ideation via changes in defeat, entrapment, and hopelessness, in a sample of eighty euthymic participants with bipolar disorder (*N* = 62 at follow-up). Linear regression models tested the extent to which perceived social support at baseline predicted changes in suicidal ideation at four months directly and indirectly via changes in defeat, entrapment, and hopelessness. Perceived social support did not directly predict changes in suicidal ideation, but there was a significant indirect mediational pathway between perceived social support at baseline and changes in suicidal ideation over time, via changes in defeat, entrapment and hopelessness, supporting the SAMS. Psychological interventions which target negative perceptions of social support early, in tandem with addressing defeat, entrapment, and hopelessness over time, present a potentially effective approach to counter suicidal ideation in people who experience bipolar disorder.

## 1. Introduction

Bipolar disorder is associated with the highest rates of suicide fatalities compared to other mental health problems [1]. It is estimated that up to 80% of people with bipolar disorder will experience suicidal thoughts during their lifetime [2,3]. Whilst numerous risk factors for suicidal thoughts and behaviors have been identified for people living with bipolar disorder [1], many are non-specific and do not clearly detail how an individual moves from thinking about suicide to engaging in suicidal behaviors [4]. In order to intervene and protect against the development of suicidal thoughts and associated distress, it is necessary to understand the specific, potentially changeable, psychological components involved in the pathways to suicidal thoughts and acts [5,6]. This is an under-researched area and was the focus of the current study.

Perceptions and feelings of hopelessness, defeat, and entrapment have been highlighted as key antecedents of suicidal experiences, particularly in prominent psychological theories of suicide such as the Cry of Pain model [7]. There is a large body of literature demonstrating that hopelessness, a feeling that the future is devoid of hope, is a strong psychological predictor of suicidal thoughts, attempts, and deaths in those with mental health problems [8,9,10], including people with bipolar disorder [11,12,13,14]. Defeat encapsulates a sense of a failed social struggle, whilst entrapment describes the perception that there is no means of escape [15,16]. A prior systematic review demonstrated that there is substantial evidence for the role of defeat and entrapment in depression, anxiety, and suicidal thoughts [17]. However, only one study to date has examined the predictive role of appraisals of defeat and entrapment in the pathways to suicidal thoughts in people with bipolar disorder. This study demonstrated that defeat predicted suicidal ideation over time although it was mediated by appraisals of entrapment [18]. Separate qualitative research has highlighted that a perceived lack of social support, often attributed to feeling defeated and entrapped by limited available support, has an important influence on the development of suicidal thoughts for people living with bipolar disorder [11]. Nevertheless, the prospective relationships between perceptions of social support with those of defeat, entrapment, and hopelessness in determining the experience of suicidal ideation have not been investigated in people with bipolar disorder.

Many contemporary psychological theories of suicidal experiences emphasize the role of perceptions of social interactions in the pathways to suicidal thoughts and behaviors. For example, the Cry of Pain model of suicide [7] has highlighted the negative influences of perceptions of having ‘no rescue’. The Interpersonal Theory has emphasized the impact of feelings of ‘not belonging’ and that one is a burden on others [19,20]. Incorporating the work of Joiner and colleagues, the Integrated Motivational Volitional model of suicide suggests that negative perceptions of social support occur most strongly at a stage following perceptions of entrapment in the pathways to suicidal thoughts and acts [21,22]. The Schematic Appraisals Model of suicide (SAMS), based on both quantitative and qualitative data, tentatively suggests that negative perceptions of social support have an impact across all stages in the pathways to suicidal thoughts and acts, but perhaps most profoundly in the early stages of the pathways to suicidal experiences by amplifying feelings and perceptions of defeat and entrapment [11,18,23].

There is a clear need to ascertain ways in which perceptions of poor social support interact with perceptions of hopelessness, defeat, and entrapment to underpin suicidal thoughts over time in people with bipolar disorder. In accordance with calls for more prospectively designed studies in this area [24] which can aid understanding of the suicidal ideation-to-enaction process [25], the question of the extent to which negative perceptions of social support impact suicidal thoughts earlier or later in the pathways to suicidal thoughts in people with bipolar disorder needs to be addressed.

Therefore, the aim of the current study was to test the extent to which a perceived lack of social support predicted suicidal ideation over time, both directly and indirectly, through changes in three mediators acting in parallel longitudinally, which were appraisals of defeat, entrapment, and hopelessness, in people living with bipolar disorder. In accord with the SAMS, it was predicted that perceptions of poor social support would lead to suicidal ideation over a four-month time period indirectly via relationships between parallel mediators of perceptions of defeat, entrapment, and hopelessness, but that direct links between negative perceptions of social support and suicidal ideation over four months would not be supported.

## 2. Materials and Methods

### 2.1. Design

A prospective longitudinal design was employed. The predictor variable was perceived social support at baseline; the mediator variables acting in parallel were changes in perceived defeat, entrapment, and hopelessness from baseline to four-month follow-up; and the outcome variable was changes in suicidal ideation from baseline to follow-up. Given the robust relationship between suicidal ideation and depression (e.g., [26]), changes of symptoms of depression between baseline and four-month follow-up were statistically controlled.

### 2.2. Participants

Eighty participants were recruited based on the following inclusion criteria:

1. Diagnosis of bipolar disorder (I or II) according to the Diagnostic and Statistical Manual of Mental Disorders (DSM) IV SCID research interview criteria [27].
2. Current euthymic mood as defined by the SCID criteria, i.e., four weeks of no clinically significant mood symptoms, prior to participating.
3. Self-reported previous experience of suicidal ideation or a previous suicide attempt.
4. Aged 18–65 years.
5. Sufficient English language skills to understand and complete the assessment measures.

Euthymic participants, who did not meet SCID criteria for a current mood episode, were sampled to limit any potential effects of the study on their wellbeing considering the suicide focus of the study and research ethics obligations.

### 2.3. Recruitment

Study referrals were sought from a number of sources, including UK National Health Service adult community mental health teams and voluntary sector organizations (e.g., Bipolar UK). Potential participants were also able to self-refer. Study advertisements were placed in local newspapers and online via social media. Participants who had self-referred could contact the first author for further information about the study.

### 2.4. Measures

#### 2.4.1. The Structured Clinical Interview for DSM-IV Axis I Disorders, Research Version (SCID)

The SCID remains the gold standard for assessing criteria for the presence of psychiatric diagnoses in clinical research trials [27]. Module A (Depressive, Manic, and Hypomanic Mood Episodes) was used to ascertain that all participants met criteria for bipolar disorder and were clinically euthymic upon entry to the present study. The co-author, RO, was fully trained in administering the SCID.

#### 2.4.2. Center for Epidemiological Studies Depression Scale (CES-D-10)

This scale assesses the presence of symptoms of depression using 10 items (e.g., ‘I was bothered by things that do not usually bother me’) within the past week using a four-point scale from ‘Rarely or none of the time (less than one day of the week)’, to ‘All of the time (5–7 days of the week)’ [28]. The CES-D-10 has comparable accuracy to the original 20 item CES-D scale in correctly identifying individuals with significant depressive symptoms (Kappa = 0.82; [29]). The scale has previously demonstrated satisfactory internal consistency reliability coefficients (Cronbach α = 0.88; [29]). The alpha coefficient in the current study was 0.87.

#### 2.4.3. The Defeat Scale

This 16-item scale measures perceptions of defeat relating to feelings of failure and low social rank (e.g., ‘I feel that I am one of life’s losers’) [15]. Participants rate the frequency of which they have experienced these perceptions during the last seven days on a five-point scale from ‘Never’ to ‘Always’. The Defeat Scale has previously demonstrated alpha coefficients between 0.86 and 0.97 [15,18,30]. The alpha coefficient was 0.96 in the current study.

#### 2.4.4. The Entrapment Scale

This scale comprises 16 items assessing the extent to which participants feel that they are trapped, both internally, i.e., within oneself, (e.g., ‘I want to get away from myself’) and externally, i.e., by external events, (e.g., ‘I have a strong desire to escape from things in my life’) [15]. For each item, participants selected a response along a five-point scale ranging from ‘Not at all like me’ to ‘Extremely like me’. The Entrapment Scale has previously demonstrated alpha coefficients between 0.93 and 0.95 [15,18,30]. The alpha coefficient was 0.94 in the current study.

#### 2.4.5. The Beck Hopelessness Scale (BHS)

This scale is designed to gain insight into participants’ perceptions about their future. There are 20 items in total, comprising both positive and negative questions, for example, ‘I have great faith in the future’ versus ‘The future seems vague and uncertain to me’ [31]. Participants indicate whether the statement applies to them within the last seven days, in this study, using a 5-point scale to optimize sensitivity. The scale has been widely used in studies with individuals who experience bipolar disorder [12,13,14]. This measure has previously demonstrated an alpha coefficient of 0.93 and a test–retest reliability of 0.85 over a period of three weeks [32]. Cronbach’s alpha for the present study was 0.94.

#### 2.4.6. The Personal Resource Scale (PRQ)

The PRQ asks participants to rate the extent to which they agree or disagree with a series of statements about their support systems [33]. The scale measures the following dimensions of social support: (1) attachment and intimacy (e.g., ‘There is someone who loves and cares about me’); (2) social integration or feelings of belonging (e.g., ‘I belong to a group in which I feel important’); and the (3) availability of advice, practical assistance and emotional support from others (e.g., ‘If I became ill, there is someone to give me advice about caring for myself’). A recent systematic review of studies using the PRQ reported that Cronbach’s alphas for the included studies ranged from 0.87 to 0.93 [34], supporting the internal consistency of the measure. The Cronbach’s alpha for the present study was 0.91.

#### 2.4.7. The Beck Suicidal Ideation Scale (BSS)

This 21-item scale measures the experience of suicidal ideation, planning, and intent within the previous week [35]. Responses lie on a three-point scale reflecting an increasing severity of suicidal ideation (e.g., ‘I have no desire to kill myself’; ‘I have a weak desire to kill myself’; ‘I have a moderate to strong desire to kill myself’). The BSS has previously demonstrated an alpha coefficient of 0.96 and test–retest reliability of r = 0.88 [36]. The alpha coefficient for the present study was 0.85.

### 2.5. Procedure

This study and the informed consent procedure received approval from the University of Manchester Research Ethics Committee and an NHS Research Ethics Committee (Reference number: 14/NW/1470). An information sheet was provided for participants who were instructed to take at least 24 h to consider the information prior to giving their informed consent. Participants were contacted by the first author by telephone and given the opportunity to ask questions about the study as part of the consent process. Following this, an appointment was arranged to complete the SCID interview. Individuals whose diagnostic eligibility had been confirmed via the SCID were sent an email link to a series of confidential online questionnaires. For those who did not have access to the internet (*n* = 14), questionnaires were posted in hard copy format with a freepost envelope to return them. After four months, participants were re-contacted and given the opportunity to take part in the follow-up assessment. For those who were interested, an email link to the confidential online follow-up questionnaires was sent or a hard copy of the questionnaires with a freepost envelope was posted as requested.

### 2.6. Data Analysis

SPSS version 22 was used to analyze the data. All variables were screened for normality in which Z-scores were calculated from the skewness and kurtosis scores divided by their respective standard errors. The cut-off criterion for normality was a Z-score greater than 1.96 or less than −1.96. All variables were standardized prior to entry into the regression models. Spearman’s rho or Pearson’s product–moment correlation coefficients were calculated based upon the normality of the distribution of the data for each variable. To test for mean differences in key variables across the time points, either a paired sample t-test was used for normally distributed data, or a Wilcoxon signed-rank test was used for non-normally distributed data using the standardized test statistic.

The hypothesized mediational pathway was tested using model four of the Process algorithm for SPSS [37]. The predictor variable was perceived social support measured at baseline. The mediator variables, acting in parallel, were change scores (follow-up minus baseline) between baseline and follow-up time points in defeat, entrapment, and hopelessness. The difference in suicidal ideation between baseline and follow-up (follow-up minus baseline) was the outcome variable. The change in depression (follow-up minus baseline) across the time-points was the control variable. Direct effects and indirect effects were calculated.

Bootstrapping with 5000 random samples was used to test the significance of the indirect effects, i.e., the effect of the independent variable on the dependent variable via the mediator and/or the interaction effects of the moderator variable at 95% confidence intervals [38]. Reliable estimates of confidence intervals require at least 5000 random samples [37]. Bootstrapping offers a more rigorous alternative to Sobel’s test for analyzing the significance of mediation and moderation effects in smaller sample sizes [37].

## 3. Results

### 3.1. Sample Characteristics

The mean age of the sample was 42.80 years (SD = 12.25; range = 22–65). The follow-up assessment was completed by 62 of the original 80 baseline participants (78%). Of the baseline sample, 63% were female (30 males; 50 females). Sixty percent of the sample were not in paid employment upon entry to the study (58% unemployed; 2% retired). In total, 43% were married or in a current relationship with a partner; 34% had never been married; 10% were separated from their previous partner; 8% were divorced; and 3% were widowed. The mean age of onset for depression was 15 years, compared to 19 years for mania or hypomania. On average, the sample had experienced 37 lifetime depressive episodes and 14 manic or hypomanic episodes.

Means, ranges, and standard deviations for the predictor variables, mediator variables, outcome variables, and controlled variables assessed at baseline and follow-up are presented in Table 1. In addition, differences between the mean or median scores across the two time points of baseline and four months were analyzed.

As displayed in Table 1, participants had lower defeat, entrapment, hopelessness, and suicidal ideation scores, and higher depression and social support scores, at follow-up compared to baseline. However, none of these differences were statistically significant. Although participants met criteria for current euthymia according to the SCID interview, the advised self-report questionnaire cut-off points indicated that participants were clinically depressed at baseline and follow-up (mean score > 10; [28]); and experiencing clinically significant suicidal ideation at baseline (mean score > 6) but not at follow-up [35].

### 3.2. Correlational Analyses

Table 2 displays the correlation coefficients for the key study variables (see Appendix A for the correlation coefficients for the relationships between the baseline and follow-up assessments). Only the change in depression scores was normally distributed. Hence, the Spearman’s rank correlation coefficient was used for all calculations. Baseline social support was significantly correlated with changes in defeat and entrapment. As might be expected, changes in suicidal ideation over time correlated with changes in defeat, entrapment, and hopelessness.

### 3.3. Test of the Predicted Mediational Pathway

As displayed in Table 3, baseline social support significantly predicted changes in defeat, entrapment, and hopelessness over time, controlling for changes in depression. Only changes in hopelessness scores significantly predicted changes in suicidal ideation scores. Social support did not have a significant direct effect on changes to suicidal ideation scores. However, baseline social support significantly predicted changes in suicidal ideation scores over time via two parallel mediators which were changes in entrapment (effect = −0.57, SE = 0.42, 95% CI = −1.83, −0.01) and changes in hopelessness (effect = 0.70, SE = 0.37, 95% CI = 0.15, 1.68), but not changes in defeat (effect = 0.54, SE = 0.42, 95% CI = −0.01, 1.74). It should, however, be noted that the non-significant pathway involving defeat may have been a function of the high correlation coefficients between defeat, entrapment, and hopelessness. When the regression model was repeated with only changes in defeat as a mediator, it predicted changes in suicidal ideation (beta coefficient = 3.40, SE = 1.14, *p* = 0.004, 95% CI = 1.10, 5.68) and the indirect effect via changes in defeat scores was significant (effect = 0.63, SE = 0.41, 95% CI = 0.08, 1.77).

## 4. Discussion

The aim of this longitudinal study was to examine the relationships over time between perceived social support, defeat, entrapment, hopelessness, and suicidal ideation in people experiencing bipolar disorder. There were two key findings. First, perceptions of social support at baseline predicted changes in perceptions of defeat, entrapment, and hopelessness over time. Second, social support at baseline had an indirect effect on changes in suicidal ideation across four months via changes in hopelessness, defeat, and entrapment. There was no direct effect of social support perceptions on changes in suicidal ideation.

Psychological mechanisms implicated in suicidal experiences as outlined by the Schematic Appraisals Model of Suicide [23] have been more widely examined in other clinical samples, including people who experience mental health problems on the schizophrenia spectrum [30], post-traumatic stress disorder [39,40,41], and depression [17,42]. The present study represents an important extension of these findings to individuals who experience bipolar disorder, and further supports the potential transdiagnostic application of the SAMS in helping to understand the development of suicidal ideation. This study is one of the few to examine psychosocial pathways to suicidal ideation in people with bipolar disorder using a longitudinal design in which temporal precedence can be examined based on a testable theory. Rather than the negative effects pertaining to social support occurring after people feel trapped and defeated as has been suggested [21], the current work demonstrates that this effect influences such perceptions of feeling defeated and trapped [23,43].

Assessing levels of perceived social support may represent an effective method of helping to identify individuals who are at a higher risk of experiencing suicidal ideation via heightened perceptions of defeat, entrapment, and hopelessness. Defeat, entrapment, and hopelessness in themselves are robust predictors of suicidal thoughts and acts [8,9,10,44]. However, understanding the factors which precipitate these feelings, in particular, a perceived lack of social support, is important for contributing to formulation-based suicide-focused psychological therapies [6,43,45]. Future research would benefit from conceptualizing and differentiating between perceptions of different types of social experiences, including social isolation, stigma, exclusion, burdensomeness, disconnectedness, loneliness, and/or social perfectionism (e.g., [46]).

There are four important clinical implications arising from these findings. Clinical approaches should aim to, first, explore individuals’ positive and negative appraisals about their social support resources in the context of experienced suicidal thoughts and acts. It should also not be assumed that an individual’s current social support resources are necessarily positive in nature because, for example, the client lives with their family. That family dynamic may feel less than supportive to the person with bipolar disorder. Second, therapeutic approaches may benefit from focusing on generating alternative escape-behaviors in response to perceived defeat and entrapment, particularly on accessing social support which is genuinely felt to be helpful during times of suicidal crisis, for instance, during the night when there is likely to be lower levels of perceived social support [42]. Third, clinical interventions might consider helping individuals to better recognize and manage the emotional states associated with underlying negative psychological perceptions, for example, by co-developing personalized formulations of the links between feeling unsupported socially and the ensuing perceptions of defeat, entrapment and hopelessness, for those living with bipolar disorder. Finally, clinical approaches could consider positively reinforcing the use of social resources which are felt to be supportive by the client throughout all therapeutic stages, but most robustly in the initial stages of therapy.

Some limitations of the present study must be acknowledged. First, the study had a relatively small sample size. Although bootstrapping was used, replication of the present study with a larger sample size would help to confirm the reliability of the findings. Second, the generalization of these empirical findings from suicidal ideation to suicidal behavior is complex because the pathways from suicidal ideation to suicide attempts and suicide deaths are not yet fully understood [5,47]. However, given that suicidal ideation is a significant risk factor for suicide attempts and death by suicide [48,49,50,51], the investigation of the perceptions of social factors which underlie the development of suicidal ideation is warranted as a potential avenue for intervention. Furthermore, since suicidal ideation is often accompanied by high levels of psychological distress, suicidal thoughts remain a highly important clinical target [6]. Third, the four-month time period in this study only allowed for two points of data collection. Whilst the present study provides empirical support showing that psychosocial factors are short-term predictors of suicidal ideation via perceptions of hopelessness, entrapment, and defeat, tracking long-term predictors over timespans of years of suicidal ideation remains an essential goal for future research. There may be additional clinical variables (e.g., medication, adherence to treatments, current mood episode status) and other psychosocial factors not measured here (e.g., stigma), which are implicated in the social support-related pathways to suicidality for people living with bipolar disorder which may be fruitful avenues for further investigation. Finally, the sample consisted primarily of middle-aged participants with established long-term experiences of living with bipolar disorder. Whether similar relationships are found amongst people with a more recent onset of bipolar symptoms and suicidal experiences would be of interest in future research.

## 5. Conclusions

This study is the first to examine the relationships over time between perceived social support and suicidal ideation, as hypothesized by the Schematic Appraisals Model of Suicide (SAMS) [23], with a sample of people living with bipolar disorder. The findings make an important theoretical contribution because they support an indirect pathway from negative perceptions of social support to suicidal ideation via perceived defeat, entrapment, and hopelessness over time, consistent with the predictions from the SAMS [23]. These findings support the development of psychological interventions which target negative perceptions of social support, in tandem with appraisals of defeat, entrapment, and hopelessness over time, to reduce suicidal ideation in bipolar disorder.

## Figures and Tables

**Table 1:** Descriptive data for all assessed variables at baseline and four-month follow-up time points.

	BL Mean (SD)	FU Mean (SD)	*t* or *Z*, (*p*)	Min to Max Scores BL (FU)
**Depression**	12.94 (7.37)	14.47 (7.45)	−1.50 (*p* = 0.14)	0–26 (0–29)
**Defeat**	31.43 (16.15)	30.02 (13.52)	0.75 (*p* = 0.46)	2–63 (4–54)
**Entrapment**	29.03 (16.26)	28.12 (14.77)	0.49 (*p* = 0.63)	0–58 (4–55)
**Hopelessness**	61.27 (17.11)	57.32 (15.32)	1.86 (*p* = 0.07)	21–88 (26–90)
**Suicidal ideation**	7.27 (7.66)	5.48 (6.42)	−*1.63* (*p* = 0.14)	0–33 (0–23)
**Social support**	120.00 (27.24)	124.28 (22.89)	−1.53 (*p* = 0.13)	49–172 (69–165)

Key: BL = Baseline; FU = Follow-up. BSSI scores were analyzed with the Wilcoxon signed-rank test and the standardized test statistic is shown in italics. Analyses of the differences between the mean or median scores across the two time points are shown as *t*- or *Z*- (in italics) scores.

**Table 2:** Correlation coefficients for predictor variable, changes in the mediator/moderator variables, and the change in the outcome variable used in the regression model.

	2	3	4	5	6
**1. Baseline social support**	0.16	0.28 *	0.26 *	0.08	−0.02
**2. Defeat change score**	-----	0.71 **	0.66 **	0.38 **	0.51 **
**3. Entrapment change score**		-----	0.66**	0.25 *	0.45 **
**4. Hopelessness change score**			-----	0.36 **	0.40 **
**5. Suicide ideation change score**				-----	0.23
**6. Depression change score**					-----

* *p* < 0.05; ** *p* < 0.01.

**Table 3:** Direct and indirect effects of social support on changes in suicidal ideation via defeat, entrapment, and hopelessness.

Outcome	Predictor	Beta Coefficient	SE	*t*	*p*	95% Confidence Interval
*F* (2, 56) = 29.44, *p* < 0.00001, *R*^2^ = 0.51						
**Change in defeat**	Baseline social support	0.20	0.09	2.10	0.04	0.01–0.38
	Change in depression	0.68	0.09	7.33	0.00	0.50–0.87
*F* (2, 56) = 17.90, *p* < 0.00001, *R*^2^ = 0.39						
**Change in entrapment**	Baseline social support	0.27	0.10	2.64	0.01	0.07–0.48
	Change in depression	0.55	0.10	5.32	0.00001	0.34–0.76
*F* (2, 56) = 17.37, *p* < 0.00001, *R*^2^ = 0.38						
**Change in hopelessness**	Baseline social support	0.27	0.10	2.55	0.01	0.06–0.47
	Change in depression	0.55	0.11	5.26	0.00001	0.34–0.76
*F* (5, 53) = 4.94, *p* < 0.0009, *R*^2^ = 0.32						
**Change in suicidal ideation**	Baseline social support	0.82	0.85	0.97	0.34	−0.88–2.51
	Change in defeat	2.76	1.52	1.82	0.08	−0.29–5.80
	Change in entrapment	−2.10	1.22	−1.72	0.09	−4.55–0.35
	Change in hopelessness	2.65	1.34	1.98	0.05	−0.04–5.34
	Change in depression	−0.24	1.12	−0.21	0.83	−2.49–2.01

## Data Availability

We did not have research ethics approval or consent from the participants to publicly share their data.

## Appendix A

**Table A1:** Bivariate correlations between the key study variables at baseline and four-month follow-up.

	2	3	4	5	6	7	8
**1 BL CES-D**	*0.70* **	*0.65* **	*0.50* **	*0.52* **	*−0.37* **	*0.33* **	*−0.23*
**2 BL Defeat**		0.79 **	0.75 **	*0.54* **	−0.50 **	*0.29* *	−0.20
**3 BL Entrap**			0.78 **	*0.61* **	−0.52 **	*0.46* **	−0.30 *
**4 BL BHS**				*0.60* **	−0.40 **	*0.32* *	−0.43 **
**5 BL BSSI**					*−0.45* **	*0.53* **	*−0.35* **
**6 BL PRQ**						*−0.30* *	0.66 **
**7 FU BSSI**							−0.40 **
**8 FU PRQ**							

* *p* < 0.05; ** *p* < 0.01. Key: BL = Baseline; FU = Follow-up; CES-D = Centre for Epidemiological Studies Depression Scale; BHS = Beck Hopelessness Scale; BSSI = Beck Scale for Suicidal Ideation; PRQ = Personal Resource Questionnaire. The italicized values indicate that the Pearson’s product–moment correlation coefficient for parametric data was used, whereas the non-italicized values indicate that Spearman’s correlation coefficient was used.
